# Tackling the challenges of nanomedicines: are we ready?

**DOI:** 10.1093/ajhp/zxab048

**Published:** 2021-02-18

**Authors:** John B Hertig, Vinod P Shah, Beat Flühmann, Stefan Mühlebach, Gunar Stemer, Jacqueline Surugue, Rob Moss, Tiziana Di Francesco

**Affiliations:** 1 Department of Pharmacy Practice, Butler University College of Pharmacy and Health Sciences, Indianapolis, IN, USA; 2 VPS Consulting, North Potomac, MD, USA; 3 Vifor Pharma Ltd, Glattbrugg, Switzerland; 4 Division of Clinical Pharmacy & Epidemiology and Hospital Pharmacy, Department of Pharmaceutical Sciences, University of Basel, Basel, Switzerland; 5 Pharmacy Department, Vienna General Hospital–Medical University Campus, Vienna, Austria; 6 Hospital Pharmacy Department, Georges Renon General Hospital, Niort, France; 7 Hospital Pharmacy Section, International Pharmaceutical Federation, The Hague, Netherlands

**Keywords:** nanomedicine, nanosimilars, pharmacists, substitution, therapeutic equivalency

## Abstract

**Purpose:**

This review provides an overview of the proceedings of the symposium “Tackling the Challenges of Nanomedicines: Are We Ready?” organized by the International Pharmaceutical Federation (FIP) Hospital Pharmacy Section and Non-Biological Complex Drugs (NBCDs) Working Group at the 2019 FIP World Congress of Pharmacy and Pharmaceutical Sciences. Debate centered on reasons underlying the current complex regulatory landscape for nanomedicines and their follow-on products (referred to as nanosimilars) and the pivotal role of hospital pharmacists in selecting, handling, and guiding usage of nanomedicines and nanosimilars.

**Summary:**

The evaluation and use of nanomedicines are recognized among scientific, pharmaceutical, and regulatory bodies as complex. Interchangeability and substitutability of nanomedicines and nanosimilars are confounded by a lack of pharmaceutical and pharmacological equivalence, reflecting the inherent complex nature of these drug products and manufacturing processes. Consequences include implications for clinical safety and efficacy and, ultimately, comparability. Local regulatory approvals of some nanomedicines have occurred, but there is no standard to ensure streamlined evaluation and use of consistent measures of therapeutic equivalence of reference products and their nanosimilars. Hospital pharmacists are expected to be experts in the selection, handling, and substitution of nanomedicines and familiarize themselves with the limitations of current methods of assessing pharmaceutical and clinical equivalence of nanosimilars in order to ensure informed formulary decision-making and eventual patient benefit.

**Conclusion:**

Supportive guidance for pharmacists focusing on the substitutability and/or interchangeability of nanomedicines and their nanosimilars is needed. Current FIP guidance for pharmacists on therapeutic interchange and substitution should be extended to include nanomedicines and nanosimilars.

**KEY POINTS**

- Interchangeability of reference nanomedicines and their follow-on products is not supported by well-defined equivalence evaluations; growing evidence shows adverse clinical and cost implications with use of nanosimilars.
- For patient safety and benefit, regulatory approval processes for nanosimilars need to be better defined and standardized across agencies to support consistent evaluation of critical quality attributes as evidence of therapeutic equivalence to reference nanomedicines.
- Pharmacists would benefit from additional education and guidance on interchangeability of nanosimilars; in support, current International Pharmaceutical Federation guidance on therapeutic interchange and substitution should be updated.

A large and steadily growing body of literature focuses on nanomedicine and the challenges of the interchangeability or substitution of nanomedicines and their follow-on products, also referred to as nanosimilars.^1-3^ In September 2019, the International Pharmaceutical Federation (FIP) Hospital Pharmacy Section^4^ and the FIP Non-Biological Complex Drugs (NBCDs) Working Group^5^ organized a precongress satellite symposium at the 79th FIP World Congress of Pharmacy and Pharmaceutical Sciences in Abu Dhabi, United Arab Emirates.^6^ Chaired by authors Shah and Hertig, the symposium included presentations by 4 speakers (authors Flühmann, Mühlebach, Shah, and Stemer) that provided scientific, industrial, clinical practice, and hospital pharmacy perspectives on current challenges in nanomedicine and appropriate use and evaluation of nanosimilars. Here we provide an overview of the topics covered and discussed during the session, which emphasized the importance of promoting wider understanding of nanomedicines and the innovation behind them. Those topics included consideration of the challenges arising from the potential impact of disparities between reference nanomedicine products and nanomedicines to ensure their safe and effective use in clinical practice. Presentations explored differences between nanomedicines and conventional small-molecule drugs and reasons for the current complex regulatory landscape for nanomedicines and nanosimilars. Finally, the symposium analyzed the pivotal role of hospital pharmacists in decisions on product selection and usage due to their expected biomedical understanding of drugs’ efficacy and safety profiles and their impact on patient treatment. Debate led to the conclusion that there was a need for revision of the 2018 FIP policy document “Pharmacist’s Authority in Product Selection: Therapeutic Interchange and Substitution” to include guidance on nanomedicines and their follow-on products.^7^

## What are nanomedicines?

There is no universally accepted definition of nanomedicine. The term *nanomedicine* describes the use of nanotechnology in biomedical science and healthcare and encompasses a wide range of therapeutic and diagnostic applications.^1,2^,^8^ The European Medicines Agency (EMA) designates nanomedicine as “the application of nanotechnology in view of making a medical diagnosis or treating or preventing diseases” through exploiting the properties of materials at nanometer scale (approximately 0.2-100 nm).^9^ In the United States, the Food and Drug Administration (FDA) follows a more restrictive approach, considering both size (materials with nanoscale dimensions of approximately 1-100 nm) and function (whether physical or chemical properties or biological effects are attributable to dimensions up to 1,000 nm) to determine whether a product involves nanotechnology.^10^ In 2017, FDA issued a draft guidance on drug products that contain nanomaterials.^11,12^

Nanomedicines are already a reality of modern healthcare. A PubMed search for “nanomedicine” in April 2020 retrieved 30,637 results. In a review of nanomaterial submissions to FDA from 1973 through 2015, 20 different categories of nanomaterials were listed,^13^ with nanoparticle structures ranging from solid or functionalized nanoparticles to nanoshells, nanotubes, and nanoliposomal vesicles.^14^ Nanomedicines have already been applied to a broad spectrum of medical specialties, often for chronic and severe diseases—for example, iron deficiency anemia,^15,16^ fungal infections and leishmaniasis,^17,18^ chronic dry eye disease,^19^ cancers,^20,21^ and multiple sclerosis.^22,23^ Liposomes, nanocrystals (introduced mainly for increasing the solubility/bioavailability of oral drugs^24^) and iron-carbohydrate complexes comprise more than two-thirds of nanomedicine product submissions to FDA.^13^ There is also increasing discussion surrounding nanomedicines and their integration into healthcare, a theme explored in a June 2018 webinar series presented by the hospital pharmacy section of FIP.^25-27^

## Why “go nano” in therapeutics?

**Figure 1.**
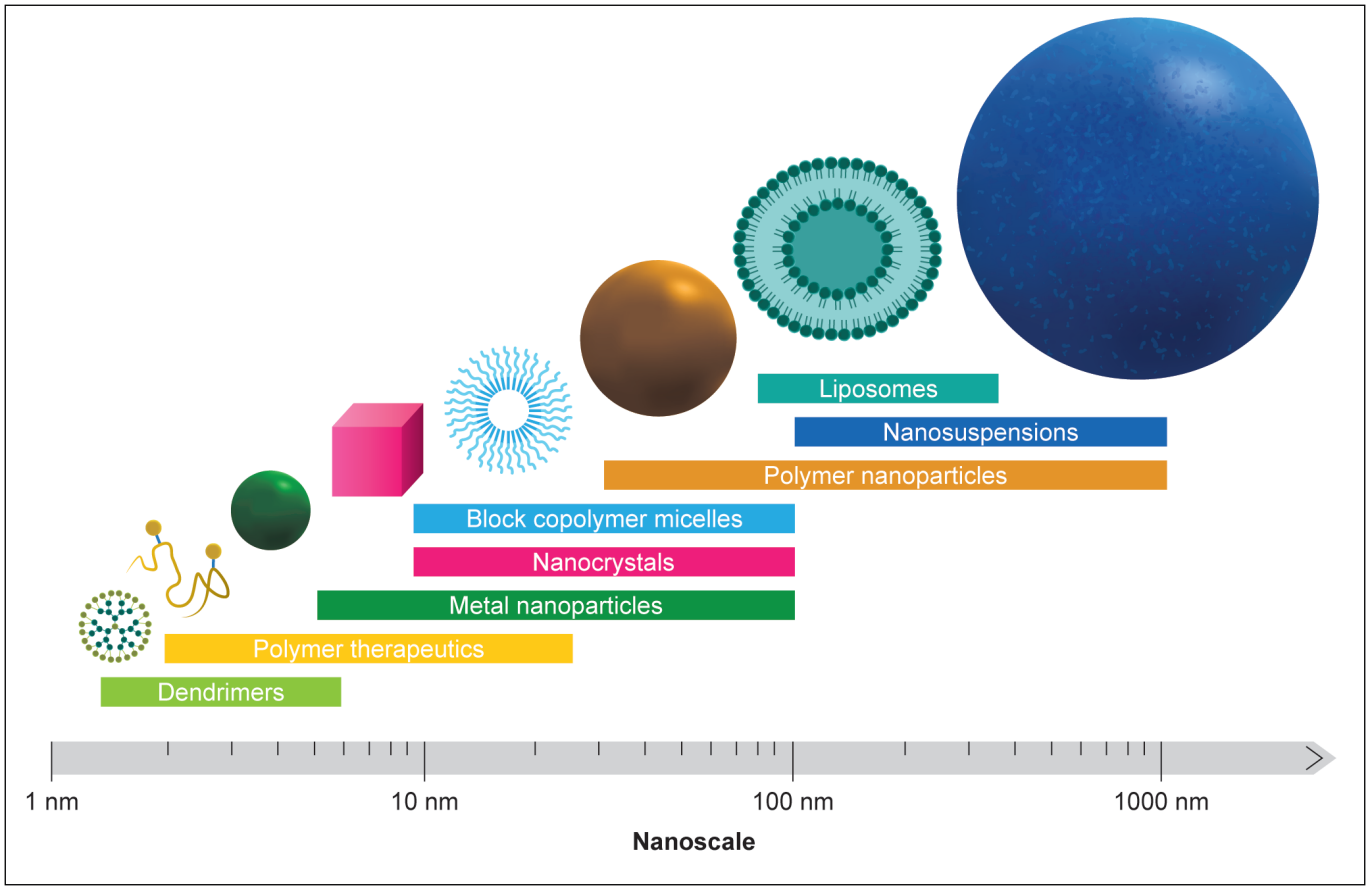
Different surface structures of nanomedicines, which can affect their interaction with the biological environment. Adapted, with permission, from an original figure developed by Dr. Tom McDonald (University of Liverpool) for the British Society for Nanomedicine.

Almost all materials can be nano-sized. The reduction in particle size gives rise to specific properties that differentiate nanomedicines from other drug products.^10,14^ One determinant of the fate of nanomedicines upon administration is the interaction of their surface with the biological environment (Figure 1). Nanoparticle size, size distribution, morphology, and surface characteristics all influence drug delivery, pharmacokinetics, and pharmacodynamics as well as the toxicity and immunogenicity profiles of a drug product.^28,29^ Interactions with biological systems, such as translocation routes into cells or reactivity with cells and cellular components, are also impacted.^30^ For example, nanonization and surface modification can impact the biodistribution of colloidal drugs, altering cellular and organ-level uptake to allow selective or preferential drug targeting,^29,31^ thus impacting pharmacokinetics.

The particular properties of nanomedicines associated with their nanoscale dimensions can lead to certain therapeutic advantages. For example, paclitaxel protein-bound particles (Abraxane, Celgene Corporation, Summit, NJ) are a type of nanomedicine, comprising specifically engineered paclitaxel-loaded albumin moieties, licensed for use for several oncology indications, including metastatic non–small cell lung carcinoma.^20,21^ They were developed as a solvent-free taxane-based medicine to overcome hypersensitivity reactions and toxicity associated with a previous formulation in which amphiphilic solvents were used to solubilize lipophilic paclitaxel.^32^ Preclinical evidence suggests that the specific albumin-mediated and stabilized colloidal formulation of paclitaxel protein-bound particles could also facilitate penetration of the blood-brain barrier to enable targeting of brain tumors.^33,34^ Polyethylene glycol (PEG) conjugation is another strategy that may offer benefits in nanomedicine delivery. In an example of use of this technology, PEG-conjugated liposomal doxorubicin (pegylated liposomal doxorubicin hydrochloride [Doxil/Caelyx; Janssen Biotech, Inc., Horsham, PA]) improves plasma stability and half-life compared with either nonpegylated liposomal doxorubicin (Myocet liposomal; Teva Pharmaceutical Industries Ltd, Petah Tikva, Israel) or free doxorubicin (Adriamycin; Pfizer Inc., New York, NY). The higher in vivo stability of PEG-conjugated nanomedicine results in an increased tumor exposure with a lower total dose and, as a consequence, reduced toxicity (associated with free and total-dose doxorubicin) relative to the other doxorubicin formulations, increasing its efficacy and thereby improving the therapeutic index.^28,35^,^36^

## Challenges in demonstrating therapeutic equivalence of nanomedicines and follow-on products

Nanomedicines are complex in composition and structure and will therefore also fall into the pharmaceutical class of *non-biological complex drugs* (NBCDs)—a term coined in 2012 based on a concept first arising at a workshop in 2009.^37,38^ NBCDs are considered to be complex drugs that are not biologics but, in common with biologics, lack a homogeneous molecular structure that cannot be fully isolated or characterized on the basis of chemical structure alone.^39^ Instead, a specific set of orthogonal physicochemical analytical methods must be applied and complemented in a weight-of-evidence approach with nonclinical and human data to support similarity with a reference product.^40^ While neither FDA nor EMA currently accept NBCDs as constituting a distinct category of drugs, FDA refers to “complex” drug products on the basis of factors including the complexity of the active ingredient, formulations, delivery routes, and dosage forms.^41,42^ Examples of NBCDs include liposomes,^17,18^ emulsions,^19^ glatiramoids,^22,23^ and iron-carbohydrate complexes,^15,16^ as well as albumin-bound nanoparticle anticancer drugs.^20,21^ In addition, nanomedicines are very sensitive to changes in manufacturing process conditions and production scales, which can affect a product’s quality and clinical profile, including batch-to-batch consistency.^12,43^ With the quality and composition of nanomedicines highly dependent on sophisticated and difficult-to-control proprietary manufacturing processes, even small differences in process conditions may lead to differences in their critical quality attributes (CQAs).^1^ A CQA is defined by the International Council for Harmonisation of Technical Requirements for Pharmaceuticals for Human Use as “a physical, chemical, biological, or microbiological property or characteristic that should be within an appropriate limit, range, or distribution to ensure the desired product quality,” and CQAs must be defined for each nanomedicine. As of today, structure-function relationship is not fully understood for many nanomedicines, and hence product-specific CQAs might be ill defined for many approved drug products; this is a topic subject to ongoing scientific debate.^24^

Given the complexities of characterizing nanomedicine structures as well as the limitations of standard assays, test protocols, and laboratory instrumentation,^8^ it is extremely challenging to fully demonstrate the pharmaceutical equivalence of follow-on products to reference nanomedicines. A report of the US Government Accountability Office indicated general agreement among representatives from FDA, manufacturers, and expert groups that demonstrating pharmaceutical equivalence and bioequivalence of follow-on products to a reference nanomedicine is difficult.^41^ Given these challenges, it is inappropriate to apply the currently defined generics paradigm to nanomedicine regulation, which requires determining both pharmaceutical equivalence and bioequivalence in a comparability process for fully characterized small-molecule drug products.^24,41^ In addition, the complex nature of nanomedicines and the known barriers in demonstrating therapeutic equivalence challenge the substitutability and interchangeability of reference and follow-on products.^1,43^ Conventional bioequivalence testing (ie, measuring plasma drug concentrations) may not reflect the performance of nanomedicines, depending on administration route and different cellular uptake mechanisms in target tissues; as such, additional testing such as clinical studies may be required.^41^ Furthermore, given the sensitivity of nanomedicines to manufacturing conditions, specific, well-controlled, and robust manufacturing processes are fundamental to defining the product profile, quality, safety, efficacy, and consistency of nanosimilars.^24,43^

FDA and EMA differ in their nomenclature and regulatory approach to nanosimilars, although both regulatory bodies recognize and are considering them in their guidance.^41,44^,^45^ FDA, for example, is supporting the development of product-specific guidance for the development of “complex generic drug products.” ^41^ In terms of regulatory pathways, US and European Union (EU) policies are not harmonized and, as yet, there are no mutually accepted approval pathways for complex generic drug products and nanosimilars, respectively^46^; in fact, both the United States and EU evaluate these drug products on a case-by-case basis.^11,42^ Furthermore, EU regulatory application procedures have been noted to have been used inconsistently even within product classes.^42^

FDA draft guidance issued in 2017 acknowledged the many challenges faced in manufacturing and approving nanomaterials, providing general recommendations on the approval of drugs containing nanomaterials and demonstration of pharmaceutical equivalence and bioequivalence for complex generic drug products.^11,12^ In addition, FDA has released product-specific draft guidance for a number of nanomedicines.^11^ For the evaluation of complex generic drug products, FDA generally adopts a weight-of-evidence approach requiring stepwise and case-by-case comparison to a reference drug.^40^ The 505(j) abbreviated new drug application pathway, recommended by FDA as the standard evaluation pathway for complex generic drug products, including those comprising nanomaterials, allows approval of a generic drug based on bioequivalence to the reference product.^47^ This pathway involves designation of the complex generic drug product as pharmaceutically equivalent and bioequivalent, enabling marketing of the product as substitutable with the reference product.^12^ However, several experts have highlighted the problematic nature of this approach.^47^ Application sponsors have the option of using the 505(b)(2) pathway instead to establish the clinical safety and effectiveness of a given complex generic drug product.^12^ Nevertheless, substitutability or interchangeability with a (closely) related reference drug remains unclear in the absence of clinical head-to-head analysis.

In Europe, nanomedicines have no dedicated regulatory pathway and, unlike biologics, can be approved via the decentralized EMA procedure, despite their complexity.^42^ In the past, nanosimilars marketed in EU member states have been approved using the generics pathway.^42^ Since 2015, a totality-of-evidence approach to regulatory approval of nanosimilars has been increasingly adopted by EMA, which uses the so-called hybrid pathway authorized in Article 10(3) of Directive 2001/83/EC.^42^ In addition, EMA has released several reflection papers regarding selected nanomedicines and their nanosimilars, such as iron-carbohydrate complexes and intravenous (IV) liposomal products, in order to address specific challenges and data requirements for particular products.^48,49^ These reflection papers detail the regulatory requirements for these products, including data requirements based on a stepwise totality-of-evidence approach^44,48^,^49^ similar to the biosimilar approval pathway.^50^ In the case of nano-sized colloidal IV iron-carbohydrate complexes, the approval of a similar product currently requires more than the conventional demonstration of bioequivalence to the reference nanomedicine, such as through establishing comparable plasma iron concentrations.^48^ Indeed, the stability of the iron-carbohydrate complex as well as its physicochemical properties has a strong influence on the in vivo fate and resulting toxicological and pharmacological effects, which must also be shown to be equivalent. This necessitates the provision of sufficient evidence of product quality, including the composition of the carbohydrate matrix, spectroscopic properties, amount of labile iron released from the administered product, impurities, morphology, particle size, size distribution, charge, and surface properties, as well as the degradation pathway for the iron-carbohydrate complex.^48^ In addition, the pertinent EMA reflection paper^48^ recognizes the limitations of quality characterization for nanomedicines, noting that this alone is insufficient to provide assurance of similarity between a reference product and proposed nanosimilar. Instead, data from quality, nonclinical, and human pharmacokinetic studies are required to support regulatory approval, with a potential for requirement of additional clinical studies.^48^

Important issues remain to be addressed by both regulatory agencies, including the interchangeability and substitution of nanomedicines and nanosimilars. Therapeutic equivalence of 2 products enables them to be interchanged. Interchangeability can be either at a population level, meaning that both products can be used to treat the same condition in the same population, or at the individual level, meaning that 2 products can be switched during treatment of an individual patient.^51^ Substitution is a policy that allows for replacement, at the individual level, of a medicinal product with a similar bioequivalent product.^51^ Some have expressed the view that recent FDA draft guidance on complex generic drug product approval pathways should be reconsidered, as the agency’s proposals do not adequately account for the complexity of nanomedicines.^47^ Currently, EMA does not define substitution or interchange of follow-on products; this is decided at a national authority level by individual EU member states through application of heterogeneous and unclear rules.^24,42^,^51^ Indeed, challenges and deficiencies encountered in past approval processes have been highlighted by reports in the literature of lack of therapeutic equivalence and safety issues with some follow-on iron-carbohydrate complexes,^52,53^ as discussed in depth later in this article. Recently, a nanosimilar regulatory pathway more aligned with biosimilar approval pathways has been proposed,^3,47^ with proponents suggesting the need for a comprehensive totality-of-evidence approach of stepwise physicochemical characterization, nonclinical studies, and clinical comparative studies. Potentially, this could offer an alternative, more appropriate regulatory pathway for nanosimilar follow-on products.

## Nanosimilar selection and substitution practice: the role of the pharmacist

The 2011 “Joint FIP/WHO Guidelines on Good Pharmacy Practice” recommend that pharmacists are central to generic substitution.^54^ Hospital pharmacists also have a key role in the selection, handling, and substitution of nanomedicines, a class of drugs distinct from generics. Taking into account that there are US and EU regulatory pathways that do not establish therapeutic equivalence of follow-on products with their reference drugs, pharmacists need to be able to critically appraise the data for nanosimilars before making science-based recommendations for their inclusion in a hospital formulary; this includes familiarity with the concept of pharmaceutical equivalence (ie, quality, including purity, physico- and immunochemical properties; biological activity; and formulation characteristics) and bioequivalence (ie, comparability of pharmacokinetics as a prerequisite for clinical efficacy and safety).^43,55^,^56^ Alongside consideration of other criteria, decisions on formulary inclusion must be based on evidence of therapeutic equivalence in a relevant clinical setting.^55^

**Figure 2.**
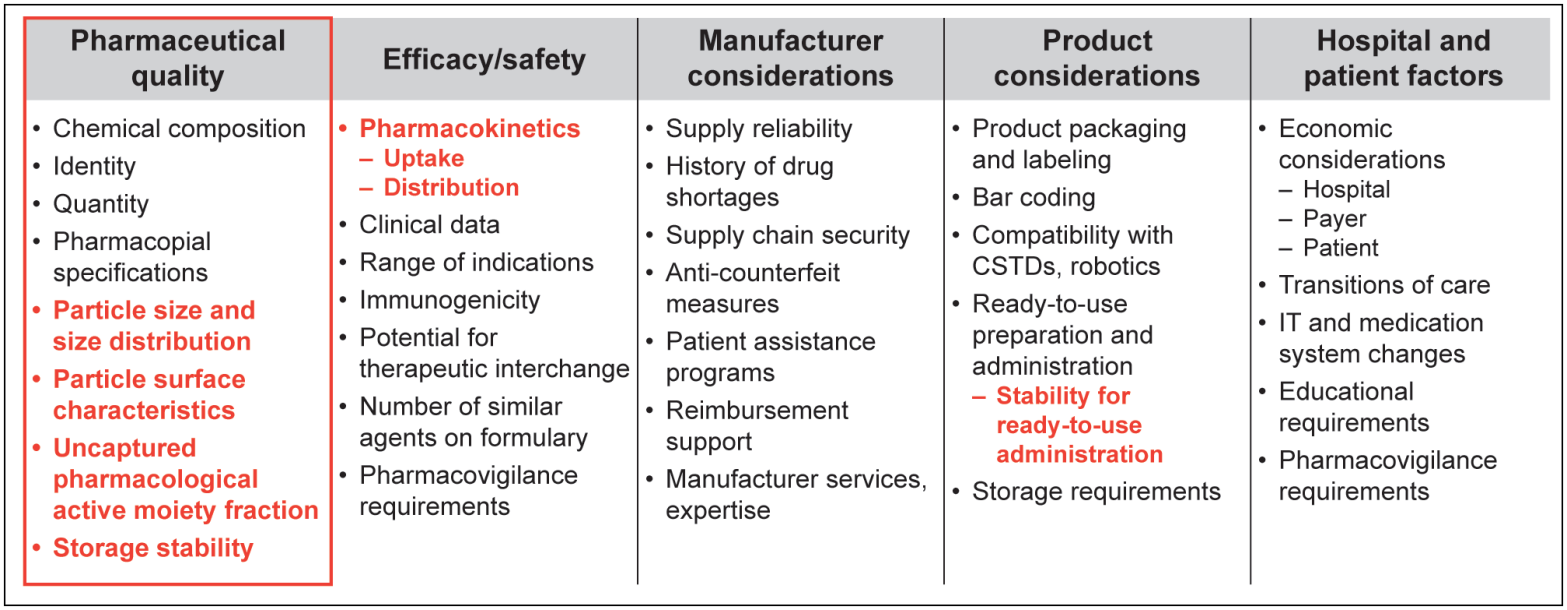
Formulary selection criteria for nanosimilars. CSTD indicates closed-system transfer device; IT, information technology. Reproduced, with permission, from reference 55.

In 2017, an expert group identified specific formulary inclusion criteria for the evaluation of nanosimilar follow-on products and developed a structured tool to guide pharmacists’ evaluation of interchangeability and substitutability (Figure 2).^55^ The following properties were considered key: particle size and particle size distribution, particle surface characteristics, the fraction of uncaptured bioactive moiety, the physical stability of the colloidal dispersion during storage and the stability of ready-to-use preparations (ie, on dilution), and uptake and distribution.^55^ Without better understanding of the limitations of interchangeability and substitution of nanomedicines, the formulary evaluation of nanosimilars may be limited to an analysis of procurement cost only, at the potential expense of safe and efficacious use of these drugs.^56^ For biologicals, interchangeability is usually decided at the physician prescribing level and is a prerequisite for substitution policies that allow for replacement of a prescribed medicine at the pharmacy dispensing level.^51^ Given that the same practice patterns may be adopted for the interchange and substitution of nanomedicines, guidance and education are needed to guide pharmacists and other healthcare professionals involved in formulary decision-making regarding nanomedicines.^43,55^,^56^

In 2018, FIP published a statement of policy outlining the pharmacist’s role and authority in product selection.^7^ The document focused on therapeutic interchange and substitution and included biologicals and biosimilars.^7^ Guidance on nanomedicines and nanosimilars was not included. Given the growing availability and use of nanomedicines, there is a clear need to revise and update this document to reflect the challenges in their evaluation.

## Implications of nanosimilars for pharmaceutical practice: the cases of liposomal doxorubicin, paclitaxel, glatiramoids, and iron sucrose

As discussed, the therapeutic equivalence of nanomedicines and their nanosimilars cannot be assured by pharmaceutical equivalence and bioequivalence due to the difficulties associated with their full characterization.^55^ The uncertain relationship between liposomal composition and clinical effect has been noted for liposomal doxorubicin formulations, raising questions as to the feasibility of introducing follow-on products.^57^ However, in 2012, due to a shortage of doxorubicin hydrochloride liposome injection (Doxil, Janssen Biotech, Inc., Horsham, PA), FDA authorized the temporary importation of a “generic” nanosimilar liposomal formulation (Lipodox, Sun Pharmaceutical Industries Ltd, Mumbai, India).^58^ Per the product labels, both the reference doxorubicin liposomal injection product (Doxil) and its nanosimilar contain the same doxorubicin active ingredient and composition of liposomes.^59,60^ Currently, 2 different nanosimilar formulations of doxorubicin hydrochloride liposome injection are approved in the United States (one manufactured by Sun Pharmaceutical Industries and one by Dr Reddy’s Laboratories Inc.).^61^ The FDA review letters for the 2 nanosimilar doxorubicin liposome products cite approval based on the demonstration of bioequivalence to the reference product.^62,63^ In contrast, in 2011 Sun Pharmaceutical Industries Europe B.V. withdrew its application for marketing authorization in Europe for the same product after EMA deemed the submitted bioequivalence and in vivo distribution studies to have provided insufficient evidence showing similarity to the reference doxorubicin hydrochloride liposome injection (Caelyx, Janssen-Cilag, Beerse, Belgium).^64^ Further data have since become available to support the similar physicochemical properties, in vivo toxicity, pharmacokinetics, and efficacy of nanosimilar doxorubicin liposomes (Sun Pharmaceutical) and the reference product, but clinical equivalence studies are ongoing,^65,66^ and as yet, no nanosimilar liposomal doxorubicin products have been registered by EMA.

In addition, differences in physicochemical characteristics between reference products and nanosimilars for paclitaxel protein-bound particles (Abraxane) and glatiramer acetate (Copaxone, Teva) have given rise to potential safety concerns surrounding their clinical use.^1^

**Figure 3.**
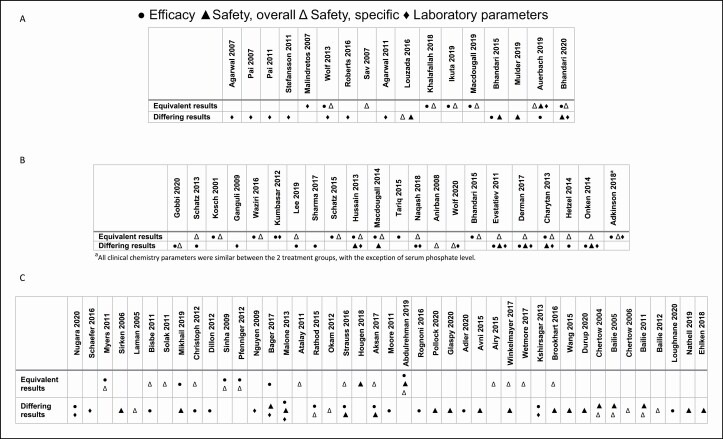
Published studies comparing efficacy and safety of parenteral iron-carbohydrate complexes: (A) head-to-head studies using the same total iron dose, (B) head-to-head studies using different total iron doses and/or regimens, and (C) retrospective studies, pharmacovigilance database studies, and meta-analyses of real-world evidence. Laboratory parameters evaluated included the following: serum ferritin, serum iron, hemoglobin, transferrin saturation, C-reactive protein, serum phosphate, mean corpuscular volume, total iron binding capacity, and the heart failure marker N-terminal prohormone of brain natriuretic peptide; these were categorized as laboratory parameters when not prespecified in a study as a primary efficacy or safety endpoint. The eAppendix provides the literature search strategy and supplemental reference list for this figure as well as detailed information on individual studies.

IV iron-carbohydrate complexes are mainly used for the treatment of iron deficiency and iron deficiency anemia. These nanomedicines comprise a polynuclear iron(III)-oxyhydroxide core stabilized by a carbohydrate shell.^1^ Designed to overcome limitations of orally administered iron(II) salts,^67,68^ several products are available in different markets (eg, iron sucrose, sodium ferric gluconate, ferric carboxymaltose, low-molecular-weight dextran, iron isomaltoside, ferumoxytol), and some follow-on versions are authorized in select countries. The in vivo drug profile of these products is influenced by the nanoparticle’s core size and the sucrose chemistry of the shell, as well as the manufacturing process and filling procedure.^1,68^ Stability affects nanoparticle distribution, bioavailability, clearance, and iron dissociation and release, influencing the safety of these products and, as a consequence, the maximum tolerated dose.^68^ The clinical performance of the different IV iron-carbohydrate complexes upon administration may vary, as reported in the literature and summarized in Figure 3. For example, IV iron sucrose has been used worldwide since the first agent in this class of drugs (Venofer, Vifor Pharma Ltd, Glattbrugg, Switzerland) was introduced in the 1950s.^1^ Evidence suggests that iron sucrose similars (ISSs), which gained access to the market as generics, differ from the reference product, resulting in varied clinical safety and efficacy and, subsequently, pharmacoeconomics.^52,53^,^69-71^ Physicochemical and preclinical studies show differences between the reference products and several ISSs in terms of oxidative stress and inflammatory responses in the liver, heart, and kidneys that may relate to the stability of the iron complex.^72-74^ In particular, ISS formulations differ from the reference product in particle size, size distribution, and visual appearance when diluted for therapeutic use.^74^ There is now considerable evidence that ISS products from different manufacturers also differ in their clinical safety and efficacy profiles.^52,53^,^69^ These findings raise questions about the interchangeability and therapeutic equivalence of ISSs and reference drug products.

The unique and complex physicochemical nature of nanomedicines and their nanosimilars necessitates strict handling, storage, and administration protocols to ensure their optimal use. Failure to adhere to specific requirements for transport and storage (eg, temperature, use of an appropriate solvent), and handling (eg, restricted dilution and speed of administration) has the potential to have negative consequences for patients.^68,69^ Notably, during the retrospective study of iron-deficient women who were postpartum or undergoing gynecologic surgeries, further dilution of ISS in an effort to reduce the frequency of adverse events in fact increased their occurrence,^69^ which may reflect the reduced stability of reactive nanoparticles.

Health-system pharmacists, including pharmacists working in acute and ambulant care as well as hospital pharmacists, are uniquely positioned to be responsible for providing insight and expertise on nanomedicine characteristics and regulatory challenges to ensure best practices in use of these innovative pharmaceuticals. To facilitate this role, pharmacists need a clear understanding of the scientific and clinical evidence underpinning decisions around substitution and/or interchange of nanomedicine reference products and their follow-on products; this highlights the important role of pharmacists in providing an in-depth scientific view on both a nanomedicine’s CQAs and their translation into patient-oriented best practice medication policies.

## Outlook for nanosimilars

The equivalence evaluation of nanomedicines is a complex and evolving, but not harmonized, area of science. Interchangeability and substitutability of nanosimilars are not clearly defined, since nanomedicine complexity leads to unknown differences in physicochemical characteristics that can translate into differences in efficacy and safety profiles between reference and follow-on products. Through use of state-of-the-art analytical techniques, two nanomedicines deemed to be of comparable physicochemical composition might be found to have differing clinical safety and efficacy profiles. This scenario illustrates shortcomings in current physicochemical characterization techniques and underscores the need for additional testing of nanosimilars to ensure that both the pharmaceutical equivalence and the safety and efficacy profiles match those of the reference product. While there is growing awareness among the scientific community, pharmaceutical industry, and both national and international regulatory bodies of the challenges presented by nanosimilar interchange, regulatory approval policies for nanomedicines still require definition, harmonization, and greater clarity to facilitate streamlined approval processes. Further requirements need to be established for the evaluation of therapeutic equivalence of nanosimilars and their reference drugs.

Health-system pharmacists are uniquely positioned as institutional experts on nanomedicine selection, handling, and substitution. Specific formulary inclusion criteria for the evaluation of nanosimilars have been published to help guide formulary decision-making.^55^ However, nanomedicines were not included in the 2018 FIP policy document outlining pharmacists’ authority in product selection on therapeutic interchange and substitution.^7^ Further supportive guidance for pharmacists focusing on the challenges of substitutability and interchangeability of reference and follow-on products is needed. We propose that the current guidance for pharmacists regarding the substitution of biologics and biosimilars should be reviewed and extended to cover the selection of nanomedicines and nanosimilars.^7^ The aim is to raise this as a topic of discussion during a combined session on nanopharmaceuticals at a forthcoming FIP World Congress of Pharmacy and Pharmaceutical Sciences.

## Conclusion

Supportive guidance for pharmacists focusing on the substitutability and/or interchangeability of nanomedicines and their nanosimilars is needed. Current FIP guidance for pharmacists on therapeutic interchange and substitution should be extended to include nanomedicines and nanosimilars.

## Supplementary Material

zxab048_suppl_Supplementary_eAppendix
