# Supplementary material for: Tackling the challenges of nanomedicines: are we ready?
Source: Am J Health Syst Pharm. 2021 Feb 18;78(12):1047–56. doi: 10.1093/ajhp/zxab048 (PMC7929390; doi:10.1093/ajhp/zxab048)
Supplement: zxab048_suppl_Supplementary_eAppendix [file zxab048_suppl_supplementary_eappendix.docx]

# eAppendix—Supplementary material

**Methods for development of eFigure 1**

A PubMed literature search was conducted to identify relevant studies on the comparative efficacy, safety, and cost-effectiveness of different intravenous iron complexes. PubMed was searched for all articles published between January 1, 2000 and May 4, 2020.

PubMed was searched using the key words “intravenous” AND “iron”, which yielded 5,454 articles in total, and 814 articles for screening after the following limits were activated:

- English language studies and articles with full-text available (i.e. abstract-only studies were excluded).
- Article type limits: Clinical study, clinical trial (incl. Phase I, II, III, IV, randomized controlled trial, controlled clinical trial, pragmatic clinical trial), meta-analysis, systematic reviews.

PubMed was also searched with the English language and full-text limits activated for “intravenous AND iron AND cost”, resulting in a further 132 results (and 54 duplicates); the medical subject heading (MeSH) term “anemia, iron-deficiency” combined with “iron” AND “intravenous”, which yielded a further 396 results (and 280 duplicates); and the free-text terms “iron-deficiency anemia AND intravenous”, which provided a further 473 results (and 799 duplicates).

After combining all the searches, the titles and abstracts of 1,815 unique articles were manually screened for articles of clinical trials, meta-analyses, retrospective or real-world studies, or cost-effectiveness studies that compared at least two intravenous iron formulations. Studies comparing intravenous iron with oral iron were not included unless there were at least two intravenous iron formulations under investigation (**Figure 3**). Additional relevant references were identified from the reference lists of selected papers.

Full-text articles were examined to determine whether the iron formulations being compared were found to be equivalent/noninferior/similar in terms of efficacy and safety, based on the definitions, statistical analyses, and/or conclusions in each original article.

### **Supplemental reference list for eFigure 1 (in order of appearance)**

A - SAME DOSING

Agarwal R et al*. Kidney Int.* 2007;72:638–642.

Pai AB et al*. Pharmacotherapy.* 2007;27:343–350.

Pai AB et al*. Biometals.* 2011;24:603–613.

Stefansson BV et al*. Nephron Clin Pract.* 2011;118:c249–c256.

Malindretos P et al*. Am J Nephrol.* 2007;27:572–579.

Wolf M et al*. J Bone Miner Res.* 2013;28:1793–1803.

Roberts MA et al*. BMC Nephrol.* 2016;17:177.

Sav T et al*. Ren Fail.* 2007;29:423–426.

Agarwal R et al*. Clin J Am Soc Nephrol.* 2011;6:114–121.

Louzada ML et al*. BMC Hematol.* 2016;16:7.

Khalafallah AA et al*. Semin Hematol.* 2018;55:223–234.

Ikuta K et al*. Int J Hematol.* 2019;109:41–49.

Macdougall I et al*. Clinical Nephrol.* 2019;91:237–245.

Bhandari S et al*. Nephrol Dial Transplant.* 2015;30:1577–1589.

Mulder MB et al*. Br J Clin Pharmacol.* 2019;85:385–392.

Auerbach M et al*. Am J Hematol.* 2019;94:1007–1014.

Bhandari S et al*. Nephrol Dial Transplant.* 2020:1–10.

B – DIFFERENT DOSING

Gobbi L et al*. Ther Apher Dial.* 2020;10.1111/1744-9987.13488.

Schatz U et al*. Atheroscler Suppl.* 2013;14:115–122.

Kosch M et al*. Nephrol Dial Transplant.* 2001;16:1239–1244.

Ganguli A et al*. Ren Fail.* 2009;31:106–110.

Waziri B et al*. Clin Kidney J.* 2016;9:817–822.

Kumbasar A et al*. J Nephrol.* 2012;25:825–832.

Lee S et al*. J Obstet Gynaecol Res.* 2019;45:858–864.

Sharma N et al*. J Obstet Gynaecol India.* 2017;67:253–257.

Schatz U et al*. Atheroscler Suppl.* 2015;18:199–208.

Hussain I et al*. Anemia.* 2013;2013:169107.

Macdougall IC et al*. Clin J Am Soc Nephrol.* 2014;9:705–712.

Tariq N et al*. J Coll Phys Surg Pak.* 2015;25:193–197.

Naqash A et al*. BMC Womens Health.* 2018;18:6.

Anirban G et al*. Ren Fail.* 2008;30:629–638.

Wolf M et al*. JAMA.* 2020;323:432–443.

Bhandari S et al*. Nephrol Dial Transplant.* 2015;30:1577–1589.

Evstatiev R et al*. Gastroenterology.* 2011;141:846–853.

Derman R et al*. Am J Hematol.* 2017;92:286–291.

Charytan C et al*. Nephrol Dial Transplant.* 2013;28:953–964.

Hetzel D et al*. Am J Hematol.* 2014;89:646–650.

Onken JE et al*. Nephrol Dial Transplant.* 2014;29:833–842.

Adkinson N et al*. Am J Hematol.* 2018;93:683–690.

C – OBSERVATIONAL STUDIES

Nugara GD *et al. Monaldi Arch Chest Dis.* 2020;90:1196.

Schaefer B et al*. PLoS One.* 2016;11:e0167146.

Myers B et al*. Br J Haematol.* 2011;153:30.

Sirken G et al*. Clin Nephrol.* 2006;66:348–356.

Laman CA *et al. J Natl Compr Canc Netw.* 2005;3:791–795.

Bisbe E et al*. Br J Anaesth.* 2011;107:477–478.

Solak Y *et al. Ren Fail.* 2011;33:307–311.

Mikhail AI et al*. BMC Nephrol.* 2019;20:13.

Christoph P et al*. J Perinat Med.* 2012;40:469–474.

Dillon R et al*. J Blood Transfus.* 2012;2012:73514.

Sinha S et al*. J Renal Care.* 2009;35:67–73.

Pfenniger A et al*. J Perinatal Med.* 2012;40:397–402.

Nguyen T. *Am J Health Syst Pharm.* 2009;66:1101–1104.

Bager P et al*. Br J Clin Pharmacol.*2017;83:1118–1125.

Malone M et al*. Obes Surg.* 2013;23:1413–1420.

Atalay H et al*. Hemodial Int.* 2011;15:374–378.

Rathod S et al*. Int J Appl Basic Med Res.* 2015;5:25–30.

Okam MM et al*. Am J Hematol.* 2012;87:e123–124.

Strauss W et al*. BMC Hematol.* 2016;16:20.

Hougen I et al*. Clin J Am Soc Nephrol.* 2018;13:457–467.

Aksan A et al*. Aliment Pharmacol Ther.* 2017;45:1303–1318.

Moore RA et al*. BMC Blood Disord.* 2011;11:4.

Abdulrehman J et al*. Transfusion.* 2019;59:3646–656.

Rognoni C et al*. Clin Drug Investig.* 2016;36:177–194.

Pollock R et al*. Expert Rev Hematol.* 2020;13:187–195.

Glaspy JA et al. *Ther Clin Risk Manag.* 2020;16:245–259.

Adler M et al*. Pharmaceuticals (Basel).* 2020;13:e85.

Avni T et al*. Mayo Clin Proc.* 2015;90:12–23.

Airy M et al*. Nephrol Dial Transplant.* 2015;30:2068–2075.

Winkelmayer WC et al*. Am J Kid Dis.* 2017;69:771–779.

Wetmore JB et al*. PLoS One.* 2017;12:e0171098.

Kshirsagar AV et al*. Am J Med.* 2013;126:541.e1–14.

Brookhart MA et al*. Am J Kidney Dis.* 2016;67:119–127.

Wang C et al*. JAMA.* 2015;314:2062–2068.

Durup D et al*. Expert Rev Hematol.* 2020;13:557–564.

Chertow GM et al*. Nephrol Dial Transplant.* 2004;19:1571–1575.

Bailie GR et al*. Nephrol Dial Transplant.* 2005;20:1443–1449.

Chertow GM et al*. Nephrol Dial Transplant.* 2006;21:378–382.

Bailie GR et al. *Arzneimittelforschung.* 2011;61:267–275.

Bailie GR. *Am J Health Syst Pharm.* 2012;69:310–320.

Loughnane F et al*. Adv Ther.* 2020;37:1218–1232.

Nathell L et al. *Drug Saf.* 2020;43:35–43.

Elken B et al. *Drug Saf.* 2019;42:463–471.

**eFigure 1.** Published studies comparing efficacy and safety of parenteral iron-carbohydrate complexes. (A) Head-to-head studies using the same total iron dose. (B) Head-to-head studies using different total iron doses and/or regimens. (C) Retrospective studies, pharmacovigilance databases, and meta-analyses of real-world evidence. FCM indicates ferric carboxymaltose; FMX, ferumoxytol; HMW-ID, high-molecular-weight iron dextran; ID, iron dextran; IIM, iron isomaltoside 1000; IPM, iron polymaltose; IS, iron sucrose; LMW-ID, low-molecular-weight iron dextran; SFG, sodium ferric gluconate; SFO, saccharated ferric oxide.

All clinical chemistry parameters were similar between the two treatment groups, with the exception of serum phosphate. Laboratory parameters include serum ferritin, serum iron, hemoglobin, transferrin saturation, C-reactive protein, serum phosphate, mean corpuscular volume, total iron binding capacity, and the heart failure marker N-terminal prohormone of brain natriuretic peptide (NT-proBNP). These are categorized under laboratory parameters when they are not prespecified in the study as a primary efficacy or safety endpoint.

Legend: ● Efficacy; ▲ Safety, overall; Δ Safety, specific; ♦ Laboratory parameters

**A**

|  | Agarwal 2007 | Pai 2007 | Pai 2011 | Stefansson 2011 | Malindretos 2007 | Wolf 2013 | Roberts 2016 | Sav 2007 | Agarwal 2011 | Louzada 2016 | Khalafallah 2018 | Ikuta 2019 | Macdougall 2019 | Bhandari 2015 | Mulder 2019 | Auerbach 2019 | Bhandari 2020 |
| --- | --- | --- | --- | --- | --- | --- | --- | --- | --- | --- | --- | --- | --- | --- | --- | --- | --- |
| Equivalent results |  |  |  |  | ♦ | ● Δ |  | Δ |  |  | ● Δ | ● Δ | ● Δ |  |  | Δ▲♦ | ● Δ |
| Differing results | ♦ | ♦ | ♦ | ♦ |  | ♦ | ♦ |  | ♦ | Δ ▲ |  |  |  | ● ▲ | ▲ | ● | ▲♦ |
| Comparators |  | | | | | | | | | | | | | | | | |
| FCM |  |  |  |  |  | * | * |  |  |  | * | * |  |  | * |  |  |
| IS | * | * | * | * | * |  | * | * | * | * |  |  | * | * |  | * | * |
| SFG | * | * |  |  |  |  |  |  | * |  |  |  |  |  |  |  |  |
| FMX |  |  |  |  |  |  |  |  |  |  |  |  | * |  |  |  |  |
| IIM |  |  |  |  |  |  |  |  |  |  |  |  |  | * | * | * | * |
| LMW-ID |  | * |  | * |  |  |  | * |  |  |  |  |  |  |  |  |  |
| HMW-ID |  |  | * |  | * | * |  |  |  | * |  |  |  |  |  |  |  |
| ID |  |  |  |  |  |  |  |  |  |  |  |  |  |  |  |  |  |
| Other |  |  |  |  |  |  |  |  |  |  | * | * |  |  |  |  |  |

**B**

|  | Gobbi 2020 | Schatz 2013 | Kosch 2001 | Ganguli 2009 | Waziri 2016 | Kumbasar 2012 | Lee 2019 | Sharma 2017 | Schatz 2015 | Hussain 2013 | Macdougall 2014 | Tariq 2015 | Naqash 2018 | Anirban 2008 | Wolf 2020 | Bhandari 2015 | Evstatiev 2011 | Derman 2017 | Charytan 2013 | Hetzel 2014 | Onken 2014 | Adkinson 2018* |
| --- | --- | --- | --- | --- | --- | --- | --- | --- | --- | --- | --- | --- | --- | --- | --- | --- | --- | --- | --- | --- | --- | --- |
| Equivalent results |  | Δ | ●Δ |  | ●Δ | ●♦ | Δ |  | ●Δ | ● Δ | ●Δ | ● | Δ |  |  | ●Δ | Δ | Δ | ● Δ | Δ | Δ | ●Δ♦ |
| Differing results | ● Δ | ● |  | ♦ |  |  | ● | ● |  | ▲♦ | ▲ |  | ●♦ | Δ | Δ♦ |  | ●▲♦ | ●▲♦ | ▲♦ | ● | ●▲♦ |  |
| Comparators |  | | | | | | | | | | | | | | | | | | | | | |
| FCM | * | * |  |  |  |  | * | * | * | * |  |  | * |  | * |  | * |  | * |  | * | * |
| IS |  |  | * | * | * | * | * | * |  |  | * | * | * | * |  | * | * | * | * | * | * |  |
| SFG | * | * | * | * |  |  |  |  | * |  |  |  |  | * |  |  |  |  | * |  |  |  |
| FMX |  |  |  |  |  |  |  |  |  |  | * |  |  |  |  |  |  |  |  | * |  | * |
| IIM |  |  |  |  |  |  |  |  |  |  |  |  |  |  | * | * |  | * |  |  |  |  |
| LMW-ID |  |  |  | * | * |  |  |  |  | * |  | * |  | * |  |  |  |  |  |  |  |  |
| HMW-ID |  |  |  |  |  |  |  |  |  | * |  |  |  |  |  |  |  |  |  |  |  |  |
| ID |  |  |  |  |  | * |  |  |  |  |  |  |  |  |  |  |  |  |  |  |  |  |
| Other |  |  |  |  |  |  |  |  |  |  |  |  |  |  |  |  |  |  |  |  |  |  |

**C**

|  | Nugara 2020 | Schaefer 2016 | Myers 2011 | Sirken 2006 | Laman 2005 | Bisbe 2011 | Solak 2011 | Mikhail 2019 | Christoph 2012 | Dillon 2012 | Sinha 2009 | Pfenniger 2012 | Nguyen 2009 | Bager 2017 | Malone 2013 | Atalay 2011 | Rathod 2015 | Okam 2012 | Strauss 2016 | Hougen 2018 | Aksan 2017 | Moore 2011 | Abdulrehman 2019 | Rognoni 2016 | Pollock 2020 | Glaspy 2020 | Adler 2020 | Avni 2015 | Airy 2015 | Winkelmayer 2017 | Wetmore 2017 | Kshirsagar 2013 | Brookhart 2016 | Wang 2015 | Durup 2020 | Chertow 2004 | Bailie 2005 | Chertow 2006 | Bailie 2011 | Bailie 2012 | Loughnane 2020 | Nathell 2019 | | Ehlken 2018 |
| --- | --- | --- | --- | --- | --- | --- | --- | --- | --- | --- | --- | --- | --- | --- | --- | --- | --- | --- | --- | --- | --- | --- | --- | --- | --- | --- | --- | --- | --- | --- | --- | --- | --- | --- | --- | --- | --- | --- | --- | --- | --- | --- | --- | --- |
| Equivalent results |  |  | ● Δ |  |  | Δ | Δ | ● | Δ |  | ● Δ | ● Δ |  | ● |  | Δ |  |  | Δ | ▲ | Δ |  | ●▲ Δ |  |  |  |  |  | Δ | Δ | Δ |  | Δ |  |  |  |  |  |  |  |  |  | |  |
| Differing results | ●♦ | ♦ |  | ▲ | Δ | ● |  | ▲ | ● | ● |  |  | ♦ | ▲♦ | ●▲♦ |  | ● Δ | Δ | ●▲ |  | ●▲ | ● |  | ● | ▲ | ▲ | ● | ▲ |  | ▲ |  | ●♦ | ▲ | ▲ | ▲ | ▲ Δ | ▲ Δ | Δ | ▲Δ | Δ | ● | ▲ | | ▲ |
| Comparators |  | | | | | | | | | | | | | | | | | | | | | | | | | | | | | | | | | | | | | | | | | | | |
| FCM | * | * | * |  |  | * |  |  | * | * |  | * |  | * | * |  | * |  |  |  | * | * | * | * | * | * | * | * |  |  |  |  |  |  | * |  |  |  |  |  | * | * | * | |
| IS |  |  |  | * |  | * | * | * | * | * | * | * | * |  | * | * | * | * | * | * | * | * | * | * | * | * | * | * | * | * | * | * | * | * |  |  | * | * | * | * | * | * |  | |
| SFG | * |  |  | * | * |  |  |  |  |  |  |  | * |  | * |  |  | * |  | * |  |  |  | * |  |  | * | * | * | * | * | * | * | * |  | * | * | * | * | * |  | * |  | |
| FMX |  |  |  |  |  |  |  |  |  |  |  |  |  |  |  |  |  |  | * | * |  |  | * |  |  | * | * | * | * |  | * |  |  | * |  |  |  | * |  | * |  | * |  | |
| IIM |  | * |  |  |  |  |  | * |  |  |  |  |  | * |  |  |  |  |  |  | * |  |  |  | * |  | * | * |  |  |  |  |  |  |  |  |  |  |  |  | * | * | * | |
| LMW-ID |  |  | * |  | * |  | * |  |  | * | * |  |  |  |  | * |  | * |  |  |  |  |  |  |  | * |  |  |  |  |  |  |  | * | * | * | * | * | * | * | * |  |  | |
| HMW-ID |  |  |  |  |  |  |  |  |  |  |  |  |  |  |  |  |  | * |  |  |  |  |  |  |  |  |  |  |  |  |  |  |  | * | * | * | * | * | * | * |  |  |  | |
| ID |  |  |  |  |  |  |  |  |  |  |  |  |  |  | * |  |  |  |  | * |  |  |  |  |  |  | * | * |  |  | * |  |  |  |  |  |  |  |  |  |  | * |  | |
| Other |  |  |  |  |  |  |  |  |  |  |  |  |  |  |  |  |  |  |  |  |  |  |  |  |  |  | * |  |  |  |  |  |  |  |  |  |  |  |  |  |  |  |  | |

*All clinical chemistry parameters were similar between the two treatment groups, with the exception of serum phosphate.

Individual studies may investigate efficacy, safety (specific and/or overall), laboratory parameter profiles alone or in combination.

In presence of discrepancies between ‘safety, specific’ and ‘safety, overall’, the study is considered to support divergence.

The majority of the head-to-head studies were designed to evaluated non-inferiority.
